# Functional Analysis of *ZmABA8ox1b* in Regulating Maize Seed Germination via ABA Catabolism and Multi-Hormone Signaling Crosstalk

**DOI:** 10.3390/plants15111685

**Published:** 2026-05-29

**Authors:** Cheng Wang, Yueming Li, Nan Hao, Lihui Sun, Nan Sun, Yanbo Wang, Yang Zhang, Shicheng Zhao, Yusheng Ye

**Affiliations:** 1 State Key Laboratory of Maize Bio-Breeding, Liaoning Academy of Agricultural Sciences, Shenyang 110161, China; cheng19850928@sina.com (C.W.);; 2 School of Pharmacy, Harbin University of Commerce, Harbin 150040, China

**Keywords:** maize, seed germination, *ZmABA8ox1b*, abscisic acid, hormone crosstalk

## Abstract

Seed germination is a critical determinant of seedling establishment, stress resistance, and final yield. ABA catabolism plays a central role in releasing seed dormancy and promoting germination, and ABA8ox is the key rate-limiting enzyme in this process. In this study, we used wild-type maize B73, and *ZmABA8ox1b* CRISPR-Cas9 knockout mutant as materials to investigate the biological function of *ZmABA8ox1b*. Compared with the wild type, the *zmaba8ox1b* mutant significantly delayed seed germination and enhanced the sensitivity to exogenous ABA. Endogenous ABA content in mutant embryos was drastically increased, indicating that *ZmABA8ox1b* is essential for ABA degradation during germination. The loss of *ZmABA8ox1b* function led to the activation of the ABA signaling pathway and severely impaired the responsiveness to exogenous ABA. Moreover, the mutation disturbed the expression and ABA responsiveness of auxin, gibberellin, ethylene, jasmonic acid, and brassinosteroid pathways, leading to a hormonal network imbalance. In conclusion, *ZmABA8ox1b* positively regulates maize seed germination by coordinating ABA catabolism and multi-hormone signal crosstalk. This study preliminarily clarifies the molecular mechanism of *ZmABA8ox1b* in germination control and provides important gene resources and theoretical support for breeding maize varieties.

## 1. Introduction

Maize is the most widely cultivated crop with the highest yield globally, serving as a staple food, feedstock, and industrial raw material. It is also a core crop safeguarding China’s food security and supporting the development of the animal husbandry and processing industries [1]. Seed germination marks the initiation of maize plant morphogenesis and represents a critical biological process linking seed development to seedling growth [2]. Germination speed, uniformity, and stress resistance directly determine the field emergence rate, seedling vigor, and population quality, ultimately affecting the maize yield and quality [3]. In agricultural production, abiotic stresses including chilling stress during spring sowing, drought, and salinity often inhibit maize seed germination, lead to uneven seedling establishment, and increase the proportion of weak seedlings [4]. Therefore, the in-depth elucidation of the molecular regulatory network underlying maize seed germination, and the identification of key regulatory genes are of great theoretical significance and guidance for breeding new maize varieties with rapid germination, high vigor, and strong stress tolerance.

Seed germination is a highly complex and precisely regulated biological process governed by multiple factors, including genetic determinants, endogenous hormones, and environmental signals [5,6]. Among these regulatory factors, the dynamic balance between abscisic acid (ABA) and gibberellin (GA) is widely recognized as the central switch controlling seed dormancy and germination [7]. As a pivotal hormone that maintains seed dormancy and suppresses germination, the endogenous ABA homeostasis in the endosperm and embryo directly governs seed dormancy release and germination initiation [8]. ABA metabolism comprises the biosynthesis and catabolic pathways, which control ABA accumulation [9]. Notably, ABA 8′-hydroxylase (ABA8ox), a rate-limiting cytochrome P450 enzyme in the ABA catabolic pathway, catalyzes the 8′-hydroxylation of active ABA into inactive or weakly active derivatives. This process lowers endogenous ABA levels, relieves dormancy constraints, and facilitates seed germination [10].

In recent years, studies in plants including Arabidopsis and rice have demonstrated that *ABA8ox* family genes play crucial roles in seed germination, dormancy maintenance, and the stress response. The knockout or silencing of *ABA8ox* genes leads to elevated endogenous ABA levels and significantly delayed seed germination, whereas the overexpression of ABA8ox accelerates ABA degradation and enhances the germination rate and stress resistance [10,11]. However, as an important cereal crop, maize harbors multiple *ABA8ox* homologs in its genome, and the functional differentiation, tissue specificity, and regulatory mechanisms of different members remain systematically uncharacterized. *ZmABA8ox1b* shows specifically and predominantly high expression during seed germination and is closely associated with endogenous ABA degradation [11]. However, its detailed expression pattern, physiological function, molecular regulatory network, and crosstalk with the ABA signaling pathway and other phytohormones during maize seed germination still lack direct experimental evidence.

Seed germination is not regulated by a single hormone but by a sophisticated and orderly network centered on ABA-GA antagonism and integrated with auxin, ethylene, cytokinin, jasmonic acid (JA), and other hormone signals [12,13,14]. Emerging evidence suggests that ABA metabolic genes not only affect their own pathway but also regulate the biosynthesis, transport, and signal transduction of other hormones through hormonal crosstalk [8,15,16]. However, it remains unclear whether *ZmABA8ox1b* modulates maize seed germination by controlling ABA homeostasis and further reshaping the multi-hormone signaling network. Additionally, maize exhibits abundant natural germplasm resources with significant genetic variation in the germination rate among different inbred lines. Whether the expression variation in *ZmABA8ox1b* contributes to this natural variation and whether it can be used as a molecular marker for rapid germination in breeding selection also need systematic investigation.

Accordingly, in this study, we used wild-type maize B73, *ZmABA8ox1b* CRISPR-Cas9 knockout mutant, and maize core inbred lines as materials. Combining the phenotypic analysis of seed germination, quantification of endogenous ABA, qRT PCR expression assay, and transcriptome sequencing, we systematically explored the biological function of *ZmABA8ox1b* in maize seed germination. Distinctively, we further clarified the specific regulatory mechanism of this gene in modulating ABA metabolic homeostasis and signal transduction, uncovered its novel regulatory role in coordinating the crosstalk among ABA, GA, JA, and other key hormone signaling pathways at the transcriptional level, and dissected the multi-hormone molecular network governing seed germination. Collectively, this study not only improves and supplements the hormone regulatory theoretical system of maize seed germination under complex internal conditions, but also provides candidate genes and theoretical references for the genetic improvement and breeding of new maize varieties with a high germination vigor, strong stress tolerance, and stable high yield.

## 2. Results

### 2.1. ZmABA8ox1b Positively Regulates Seed Germination in Maize

**Figure 1:**
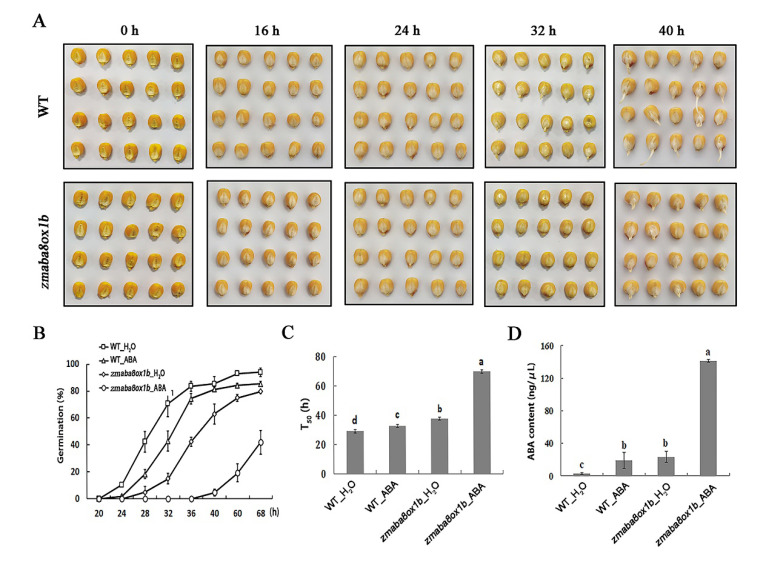
Germination phenotypes and ABA content of WT and *zmaba8ox1b* mutant seeds under H_2_O or 200 μM ABA treatment: (**A**) germination phenotypes, (**B**) cumulative germination curves, (**C**) T_50_ values, and (**D**) ABA content at 16 h after imbibition. Significance is noted at the *p* < 0.05 level. Different letters denote significant differences, while the same letter indicates no significant difference.

To investigate the function of *ZmABA8ox1b* during maize seed germination, a phenotypic analysis was conducted using the wild-type (WT) and the *zmaba8ox1b* mutant varieties. The results showed that WT seeds germinated significantly faster than the mutant (Figure 1B). As observed in the phenotypic assay (Figure 1A), at 28 h after imbibition, the germination rate of WT seeds reached 42.33%, whereas only 5% of mutant seeds germinated. The mutant exhibited a markedly delayed germination process and a significantly lower cumulative germination rate compared with WT throughout the entire test period (Figure 1B). Statistical analysis indicated that the T_50_ value was 29 h in WT but extended to 38 h in the mutant, with a significant difference between genotypes (Figure 1C). The results indicate that the *zmaba8ox1b* mutant shows significantly slower germination kinetics, confirming that functional *ZmABA8ox1b* is required for normal seed germination in maize.

### 2.2. The zmaba8ox1b Mutant Exhibits Hypersensitivity to Exogenous ABA

To investigate whether *ZmABA8ox1b* modulates ABA responsiveness during germination, the WT and mutant seeds were treated with ABA, with distilled water used as a control. As illustrated in Figure 1B, exogenous ABA significantly repressed seed germination in both genotypes. However, the inhibitory effect was far more severe in the mutant than in WT variety. Under water-treated conditions, WT seeds germinated rapidly with a T_50_ value of 29 h, whereas the germination of the mutant was delayed, resulting in a T_50_ of 38 h. Following ABA treatment, the germination of WT seeds was only moderately delayed, with the T_50_ slightly increasing to 33 h. In contrast, the mutant exhibited extreme hypersensitivity to ABA, displaying a drastically delayed germination profile and a T_50_ value extended to 70 h (Figure 1C). Collectively, these results confirm that *ZmABA8ox1b* is required for normal ABA responsiveness during seed germination. The ABA-hypersensitive phenotype of the mutant further supports that *ZmABA8ox1b* plays an essential role in regulating ABA metabolism and seed germination.

### 2.3. ZmABA8ox1b Modulates Abscisic Aid Accumulation in Maize Embryos

To further elucidate the physiological mechanism underlying the delayed germination and ABA hypersensitivity of the *zmaba8ox1b* mutant, we quantified the endogenous ABA levels in 16 h-imbibed maize embryos. Under water-treated conditions, the endogenous ABA content in WT embryos was only 2.80 ng/μL, whereas the mutant embryos exhibited a drastically elevated ABA level of 23.43 ng/μL, representing an 8.31-fold increase relative to WT. Following exogenous ABA treatment, ABA levels increased significantly in both genotypes: the WT ABA content rose to 19.11 ng/μL, while the mutant one showed an extreme accumulation of 141.26 ng/μL, which was approximately 7.4-fold higher than that of the ABA-treated WT (Figure 1D). Statistical analysis confirmed that the ABA content of the mutant under both control and ABA-treated conditions was significantly higher than that of WT, with the *zmaba8ox1b*_ABA group showing the highest ABA accumulation among all treatments. Collectively, these results demonstrate that *ZmABA8ox1b* plays an essential role in ABA catabolism during maize seed germination. The loss of *ZmABA8ox1b* function directly leads to excessive ABA accumulation in the embryos, which is the primary physiological cause of the delayed germination and ABA hypersensitivity observed in the mutant.

### 2.4. Expression Pattern Analysis of ZmABA8ox1b

**Figure 2:**
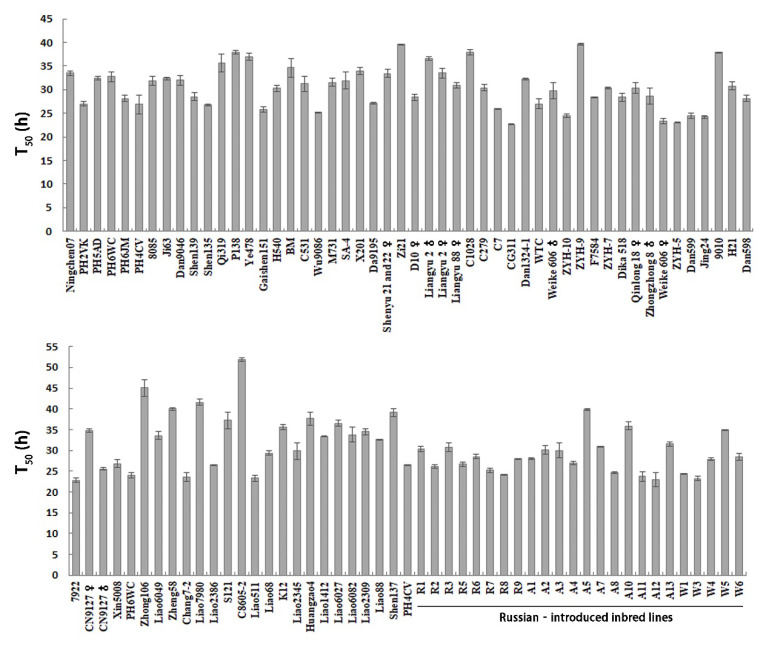
Variation in germination T_50_ among different maize inbred lines.

To elucidate the regulatory roles of genes in the ABA signaling pathway during maize seed germination, this study evaluated the T_50_ of 100 maize inbred lines, from which 15 inbred lines with significant differences in their germination speed were screened, including Liao 7922, ZHY-5, r-3, Weike 606 Female Parent, A11, R8, P138, Shen 137, ZHY-9, Zi 21, Zheng 58, Liao 7980, Zhong 106, C8605-2, and C1028. These lines covered a full phenotypic gradient of high (low T_50_), medium, and low germination rates (high T_50_) (Figure 2 and Table S1).

**Figure 3:**
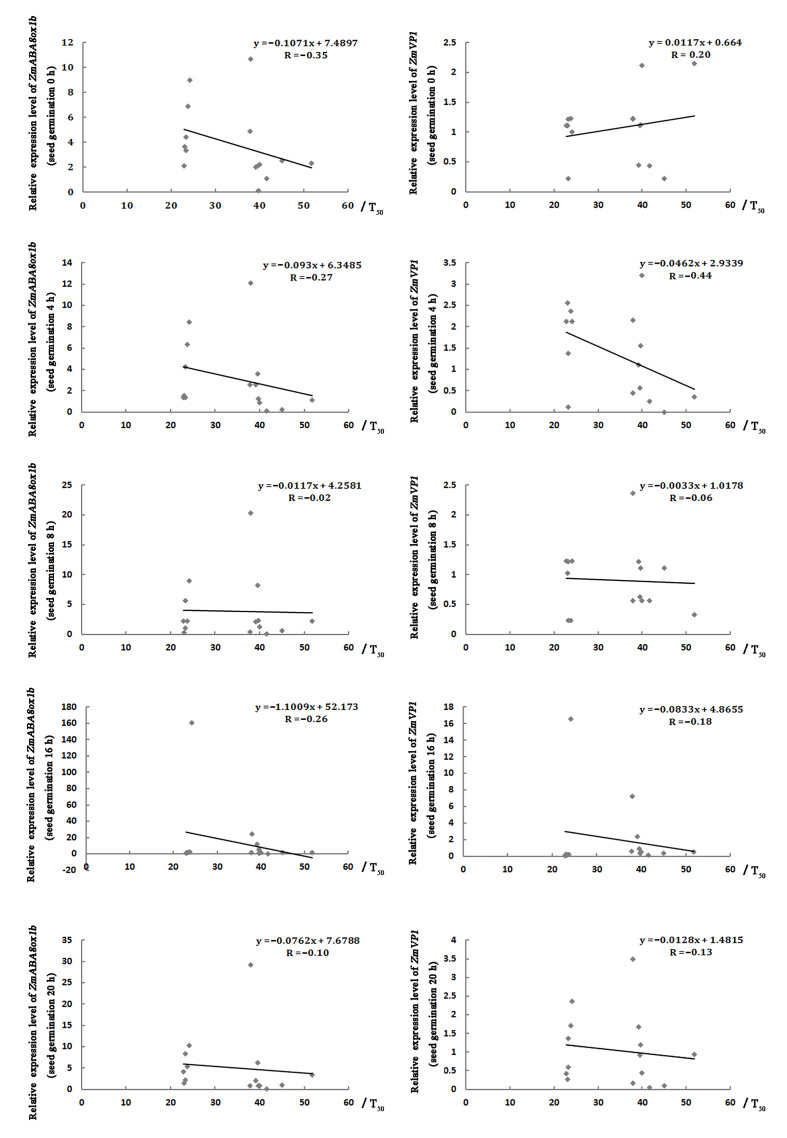
Correlation between *ZmABA8ox1b*/*ZmVP1* expression and germination T_50_.

**Figure 4:**
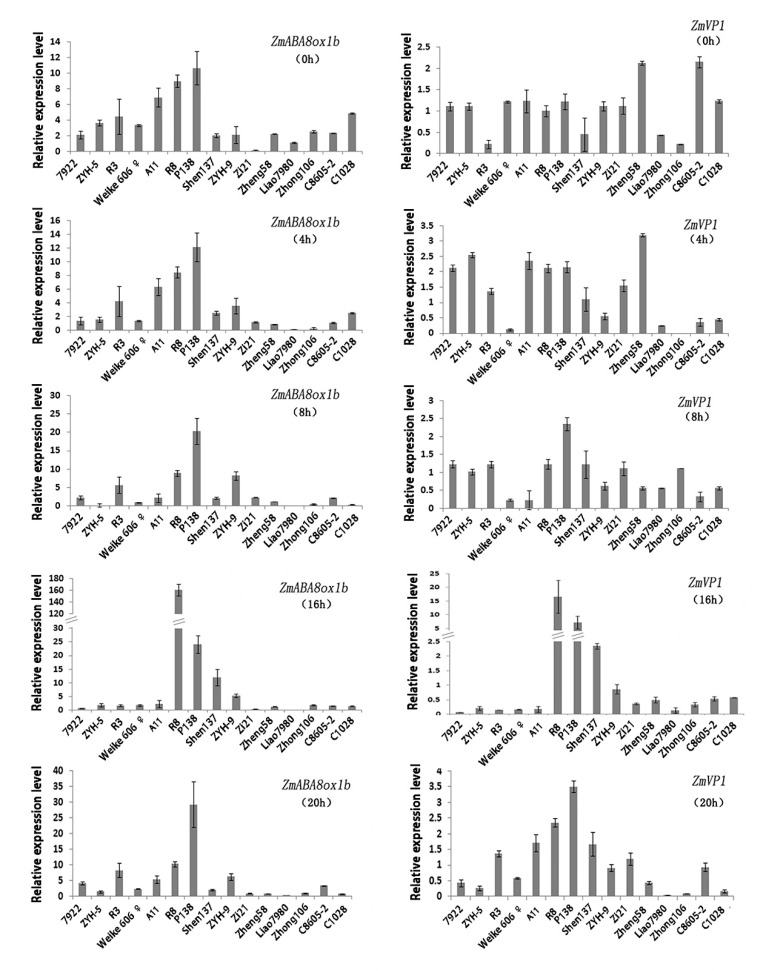
Relative expression levels of *ZmABA8ox1b* and *ZmVP1* in maize inbred lines during seed germination. *ZmActin1* was used as an internal control. Data are shown as the mean ± SD (*n* = 3).

Using the 15 selected inbred lines, the transcript levels of *ZmABA8ox1b* and *ZmVP1* [11], a key ABA-responsive gene in seed embryos, were detected by qRT-PCR at 0 h, 4 h, 8 h, 16 h, and 20 h after seed imbibition. Correlation analysis showed that the expression level of *ZmABA8ox1b* exhibited a negative correlation with T_50_ at all germination time points (Figure 3). Specifically, a decreased expression of *ZmABA8ox1b* was accompanied by a significant increase in T_50_, indicating that a low expression of *ZmABA8ox1b* markedly inhibits maize seed germination. The expression of *ZmVP1* showed a negative tendency with T_50_ at most germination stages (4–20 h), while a weak positive correlation was observed at 0 h (R = 0.20), suggesting that *ZmVP1* may be regulated by other pathways in addition to the *ZmABA8ox1b*-mediated regulatory network during seed germination. The positive correlation at 0 h likely reflects residual *ZmVP1* transcripts from seed maturation, as *ZmVP1* is highly expressed during late seed development and may persist in dry seeds, masking its germination-stage regulatory pattern. Expression analysis revealed significant genotypic variation in the transcript abundance of *ZmABA8ox1b* and *ZmVP1* among the 15 inbred lines across germination stages (Figure 4). Notably, the Russian-introduced inbred line R8 and inbred line P138 displayed a highly specific expression pattern at 16 h, a critical stage for maize seed germination; the expression levels of both *ZmABA8ox1b* and *ZmVP1* were significantly higher in these two lines than in the other 13 inbred lines. By 20 h of germination, the transcript levels of both genes decreased sharply, showing a pattern of transient high expression followed by rapid decline. In contrast, the expression profiles in the other inbred lines remained relatively stable, with a gradual upregulation during early germination (0–8 h) and steady expression after 16 h.

### 2.5. Transcriptomics Reveals the Regulatory Function of ZmABA8ox1b in ABA-Dominated Hormone Pathways

**Figure 5:**
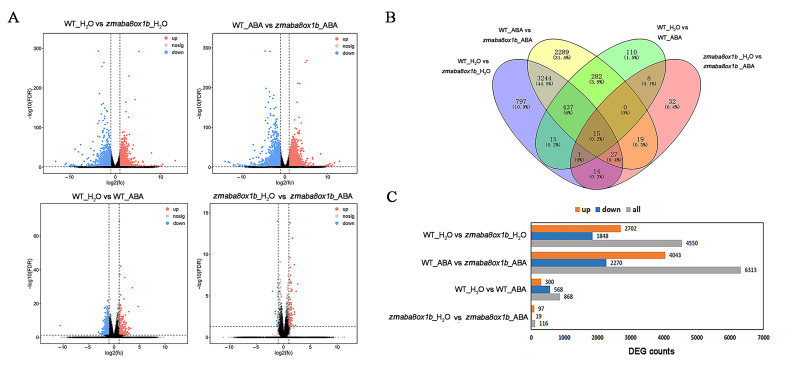
Transcriptomic comparison between wild-type and *zmaba8ox1b* mutant in response to ABA treatment: (**A**) volcano plots, (**B**) Venn diagram, and (**C**) counts of DEGs of from four pairwise comparisons.

To further reveal the regulatory mechanism of *ZmABA8ox1b* during maize seed germination, transcriptome sequencing was performed using embryos of the WT and *zmaba8ox1b* mutant at 16 h after imbibition. The results of the differential expression analysis showed significant differences in the number of differentially expressed genes (DEGs) among the four comparison groups. Under normal water conditions, a total of 4550 DEGs were identified between the mutant and the wild type, including 2702 upregulated genes and 1848 downregulated genes. After ABA treatment, the number of DEGs between the two groups increased significantly to 6313, including 4043 upregulated genes and 2270 downregulated genes, indicating that the loss of *ZmABA8ox1b* function triggered more severe transcriptional reprogramming under ABA stress. In contrast, only 868 DEGs (300 upregulated, and 568 downregulated) were identified in the wild type after ABA treatment, while the number of DEGs in the mutant after ABA treatment dropped sharply to 116 (97 upregulated, and 19 downregulated). This is likely because the mutant already exhibits constitutive ABA activation under normal (H_2_O) conditions due to the impaired ABA catabolism, such that exogenous ABA treatment cannot further trigger extensive transcriptional changes (Figure 5A,C and Tables S2–S5). A Venn diagram analysis further showed that only 15 genes were common DEGs among the four comparison groups, while 3244 overlapping DEGs existed between the WT_H_2_O vs *zmaba8ox1b*_H_2_O and WT_ABA vs *zmaba8ox1b*_ABA groups, accounting for 44.5% of the total DEGs in the two groups, indicating that the core genes regulated by *ZmABA8ox1b* are highly conserved under normal and ABA stress conditions. Meanwhile, 797 and 2289 specific DEGs were identified in the two groups, respectively, suggesting that *ZmABA8ox1b* also functions by regulating specific genes under different environmental conditions. In addition, only eight common DEGs were found between the WT_H_2_O vs WT_ABA and *zmaba8ox1b*_H_2_O vs *zmaba8ox1b*_ABA groups, further confirming that the loss of *ZmABA8ox1b* function completely altered the transcriptional response pattern of maize to ABA (Figure 5B).

**Figure 6:**
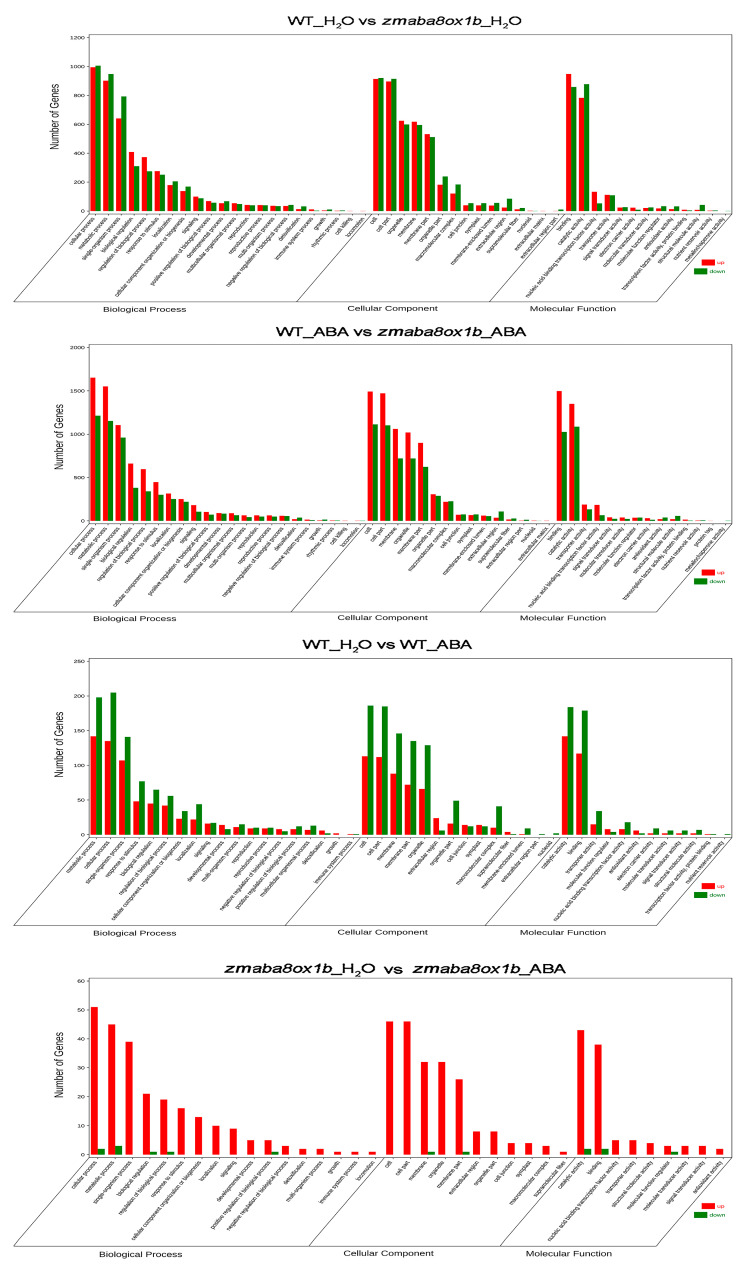
GO functional classification of DEGs in four pairwise comparisons between WT and *zmaba8ox1b* mutant in response to ABA treatment.

**Figure 7:**
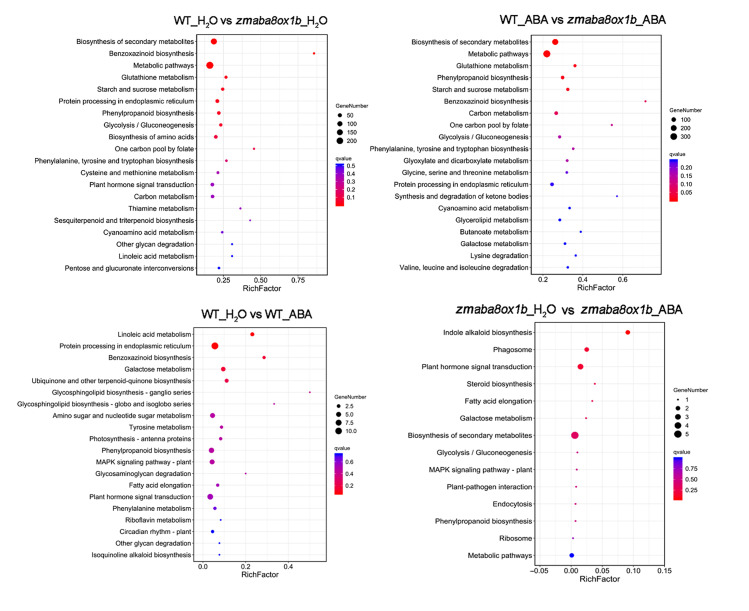
KEGG pathway enrichment analysis of DEGs in four pairwise comparisons between WT and *zmaba8ox1b* mutant in response to ABA treatment.

GO and KEGG enrichment analyses revealed highly similar enrichment patterns between the comparison groups. GO enrichment analysis revealed the functional distribution of DEGs across the three main categories. The most enriched biological process (BP) terms included cellular process, metabolic process, response to stimulus, biological regulation, and signaling. At finer GO levels, terms related to the abscisic acid response, hormone-mediated signaling, seed germination regulation, oxidation-reduction processes, and transcriptional regulation were also prominently enriched. For cellular components (CC), the dominant terms were cell, cell part, organelle, and membrane, corresponding to subcellular structures such as the nucleus, plasma membrane, and cytoplasm. In molecular function (MF), DEGs were mainly enriched in binding and catalytic activity, including sub-terms such as DNA-binding transcription factor activity, oxidoreductase activity, hormone binding, and protein kinase activity. Notably, the enrichment of stimulus- and signaling-related terms was more pronounced in the ABA-treated comparison groups, consistent with the enhanced hormone and ABA responses under exogenous ABA treatment (Figure 6). KEGG pathway enrichment analysis further confirmed that DEGs in all comparison groups were primarily enriched in the plant hormone signal transduction pathway, followed by the biosynthesis of secondary metabolites, metabolic pathways, starch and sucrose metabolism, phenylpropanoid biosynthesis, and glutathione metabolism. Notably, in both water- and ABA-treated comparison groups, the secondary metabolism and phenylpropanoid biosynthesis pathways were significantly enriched, indicating their conserved roles in the response to *ZmABA8ox1b* mutation during seed germination. In the wild type, ABA-responsive DEGs were significantly enriched in the plant hormone signal transduction and stress-related pathways, including the *MAPK* signaling pathway. In the *zmaba8ox1b* mutant, ABA treatment also induced the enrichment of the plant hormone signal transduction pathway, but the overall pattern of KEGG pathway enrichment differed notably from that of the wild type. These results indicate that the loss of *ZmABA8ox1b* alters the hormonal regulatory landscape under ABA treatment, which contributes to the inhibition of seed germination (Figure 7).

**Figure 8:**
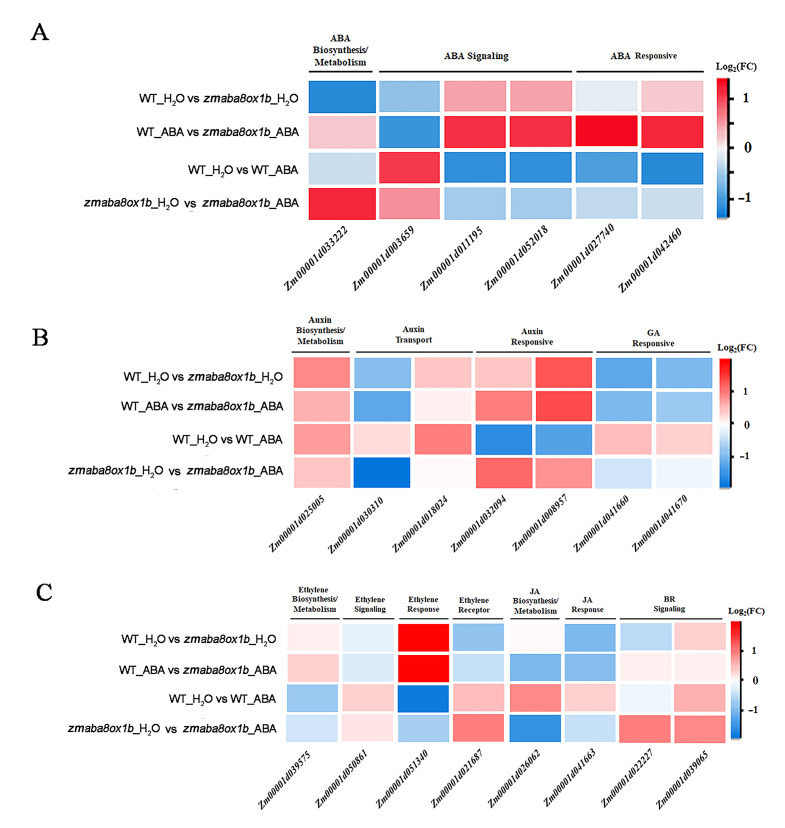
Expression profiles of key hormone-related genes in four pairwise comparisons. Heatmaps display the log_2_ (fold change) of genes associated with different hormone pathways in four pairwise comparisons. Panel (**A**) shows the ABA pathway; panel (**B**) shows the auxin and GA pathways; and panel (**C**) shows the ethylene, JA, and BR pathways.

The loss of function of *ZmABA8ox1b* resulted in the activation of the ABA signaling pathway and reduced the transcriptional responsiveness to ABA in the mutant. These expression data are derived from transcriptome analysis and listed in Supplementary Tables S2–S5. Under watered conditions, the core regulatory genes of the ABA signaling pathway exhibited a distinct expression divergence in the mutant relative to the wild type: the ABI3-VP1 family transcription factor gene *Zm00001d0424260*, the PP2C phosphatase family gene *Zm00001d011195*, and the DBP transcription factor gene *Zm00001d052018* were significantly upregulated, with log_2_(fold change, FC) values of 0.79, 1.38, and 0.69, respectively. In contrast, the key ABA biosynthetic gene *viviparous14*, and *SnRK2* were downregulated to varying degrees, with log_2_(FC) values of −0.50 and −1.85, respectively. Collectively, these expression patterns indicate the basal activation of the ABA pathway, likely arising from feedback regulation triggered by excessive endogenous ABA accumulation in the mutant. Following exogenous ABA treatment, the expression differences between the mutant and wild type were further amplified, with a markedly enhanced upregulation or downregulation of most ABA pathway genes. The upregulation of the ABI3-VP1 transcription factor gene increased from 0.79 to 2.24, that of *PP2C74* from 1.38 to 2.64, and that of *DBP1* from 0.69 to 1.45. Meanwhile, the downregulation of *SnRK2* intensified from −1.85 to −2.71. These results demonstrate that the ABA signaling pathway in the mutant is further activated under ABA stress, displaying pronounced ABA hypersensitive characteristics. In contrast, the wild type exhibited typical and normal ABA-responsive expression profiles after ABA treatment. Core ABA genes showed significant expression changes, including a prominent induced expression of the VP1 transcription factor gene and SnRK2 kinases gene, as well as responsive genes such as *rab30*, consistent with the canonical transcriptional regulation of ABA signaling in wild-type plants. However, the magnitude of the gene expression divergence between the mutant and wild type diminished following ABA treatment, indicating the attenuated responsiveness of several ABA pathway genes to ABA in the mutant, such that exogenous ABA could not further induce their expression (Table 1 and Figure 8A).

Distinct transcriptional reprogramming was also observed in other hormone pathways among the four groups. Under water control treatment (H_2_O), genes associated with auxin polar transport, including *AUX1*, were significantly downregulated in the mutant, whereas several auxin biosynthesis and early response genes showed the opposite expression trend, suggesting a reduced auxin transport capacity but relatively enhanced local biosynthesis in the mutant. Upon ABA treatment, the auxin pathway in the wild type was generally induced and activated, whereas the downregulation of transport genes was further enhanced in the mutant, with the expression changes in biosynthesis and response genes deviating sharply from those in the wild type, indicating a rewired response pattern of the auxin pathway to ABA. Genes related to GA metabolism and signaling were globally repressed in the mutant under well-watered conditions. After ABA treatment, these genes were significantly induced in the wild type but only slightly upregulated in the mutant, with a much lower induction amplitude, reflecting a severely blunted GA pathway responsiveness (Table 1 and Figure 8B). The ethylene signaling pathway was constitutively activated in the mutant. The ethylene-responsive transcription factor (*ERF* family) was highly expressed even under water control treatment. In the wild type, these genes were inhibited by ABA, whereas they remained highly expressed in the mutant and were nearly insensitive to ABA-mediated negative regulation. The JA pathway was constitutively repressed in the mutant under water control treatment, with a lower expression of the biosynthetic gene and downstream response genes compared with the wild type. Following ABA treatment, the JA pathway exhibited distinct transcriptional responses between the wild-type and the *zmaba8ox1b* mutant varieties. In the wild type, JA-related genes were significantly upregulated, indicating the activation of the JA pathway under ABA stress. In contrast, the mutant showed attenuated JA pathway responses, with most JA-related genes remaining unchanged or even downregulated. These results further indicate that *ZmABA8ox1b* regulates the JA pathway in response to ABA. For brassinosteroid (BR) signaling, two key regulatory genes were examined in this study. Under control conditions, BR signaling genes were moderately expressed in both wild-type and mutant backgrounds. Notably, ABA treatment triggered distinct responses. BR-related genes were moderately induced in the wild type but strongly upregulated in the mutant, suggesting an exaggerated activation of BR signaling upon ABA exposure. This indicates that the loss of *ZmABA8ox1b* leads to a hyperactive BR signaling response under ABA stress, further disrupting the delicate balance between ABA and BR pathways during seed germination (Table 1 and Figure 8C).

Collectively, the loss of function of *ZmABA8ox1b* disrupts endogenous ABA homeostasis, leading not only to the constitutive activation and hypersensitive response of the ABA pathway itself, but also to extensive alterations in the basal expression and ABA-responsive patterns of the auxin, GA, ethylene, and JA pathways. These changes collectively cause an imbalance in the overall hormonal regulatory network, which jointly contributes to the ABA-hypersensitive molecular phenotype of the mutant at the transcriptional level.

## 3. Discussion

Seed germination greatly affects crop stress tolerance and yield stability [17]. As a pivotal ABA metabolic enzyme, ABA 8′-hydroxylase dominates the endogenous ABA degradation process. In the present research, we clarified that the maize *ZmABA8ox1b* gene participates in seed germination regulation via modulating the ABA catabolism pathway. Further functional verification revealed that *ZmABA8ox1b* accelerates and synchronizes seed germination by balancing endogenous hormone levels, regulating ABA signal transduction, and synergistically interacting with auxin, gibberellin, ethylene, jasmonic acid, and brassinosteroid signals. Collectively, this work enriches the molecular mechanism of phytohormone-mediated seed germination in gramineous crops, and offers a valuable theoretical basis for improving maize germination characteristics.

Phenotypic analysis showed that the loss of *ZmABA8ox1b* function led to significantly delayed germination, a decreased cumulative germination rate, and prolonged T_50_. Under exogenous ABA treatment, the germination of the mutant was severely inhibited, displaying a strong ABA-hypersensitive phenotype. These observations are consistent with the functions of *AtABA8ox* in Arabidopsis and *OsABA8ox* in rice [10,11], confirming that *ZmABA8ox1b* acts as an essential positive regulator required for normal seed germination in maize. Its functional integrity directly determines whether maize seeds can rapidly break dormancy and initiate germination and early seedling growth.

Endogenous ABA accumulation was identified as the primary physiological basis underlying the mutant phenotypes. At 16 h of imbibition, a key stage for germination initiation, the ABA content in mutant embryos was 8.31-fold higher than in the wild type. Following exogenous ABA application, ABA further accumulated to much higher levels in mutant embryos than in wild-type embryos. These results demonstrate that *ZmABA8ox1b* plays a central role in ABA catabolism during maize seed germination. The loss of function of this gene directly blocks ABA degradation, leading to the abnormal accumulation of endogenous ABA, which, in turn, continuously inhibits embryo growth and cell wall loosening, ultimately resulting in delayed germination and ABA hypersensitivity. These findings are consistent with previous studies [11]. The study clarifies the functional target of *ZmABA8ox1b* at the physiological level, explains the fundamental cause of the mutant phenotype, and provides experimental evidence in maize supporting the notion that ABA metabolic homeostasis determines the rate of seed germination.

Expression analysis in diverse maize inbred lines revealed that higher *ZmABA8ox1b* transcript levels were associated with faster germination. Notably, at 16 h of imbibition, fast-germinating inbred lines such as R8 and P138 exhibited a transient peak in *ZmABA8ox1b* expression followed by a rapid decline, whereas slow-germinating lines showed a low and stable expression. This suggests that the natural variation in the spatiotemporal expression of *ZmABA8ox1b* may contribute to the divergence of the germination speed among maize germplasm, and its expression pattern could serve as a potential molecular marker for rapid germination. *ZmABA8ox1b* exerts a direct and stable regulatory role in germination, highlighting its promising value in maize molecular breeding.

Transcriptomic profiling further revealed the global regulatory framework of *ZmABA8ox1b*. Under water control treatment, thousands of DEGs were detected between the mutant and wild type. Following exogenous ABA treatment, the number of DEGs further increased, whereas the transcriptional response of the mutant to ABA was substantially attenuated. These results indicate that *ZmABA8ox1b* serves as a key regulator integrating ABA metabolism and signaling in maize. The loss of its function not only alters the basal transcriptome but also severely impairs the ABA perception and response, locking the germination regulatory network in a persistently repressed state. GO and KEGG enrichment analyses confirmed that DEGs were significantly enriched in hormone signal transduction, seed germination, redox regulation, and transcriptional control, further supporting that *ZmABA8ox1b* functions upstream in the regulatory cascade governing germination.

Within the ABA signaling pathway, the *ZmABA8ox1b* mutation triggered constitutive activation and strong feedback regulation. Genes encoding ABI3-VP1 family transcription factors (*Zm00001d042460*) and PP2C phosphatases (*Zm00001d011195*) were significantly upregulated in the mutant, whereas the gene encoding the ABA signaling pathway kinase SnRK2 was markedly downregulated. This expression pattern indicates that excessive endogenous ABA induces negative feedback, maintaining the ABA pathway in a semi-activated state and leading to the paradoxical phenotype of ABA hypersensitivity accompanied by blunted downstream responses. Exogenous ABA further amplified these changes, resulting in the more intense upregulation of VP1 and PP2C and stronger downregulation of SnRK2. These data reveal that *ZmABA8ox1b* modulates not only the ABA metabolism but also ABA signaling balance, thereby coordinately regulating seed germination through the dual control of catabolism and signal transduction. An important finding of this study is the central role of *ZmABA8ox1b* in coordinating the multi-hormone network during germination. Seed germination relies on the precise balance and dynamic crosstalk among ABA, GA, auxin, ethylene, JA, and other hormones rather than ABA action alone. Similar regulatory patterns have been reported in other plants [6,13,18,19]. The mutation of *ZmABA8ox1b* led to multiple hormonal disorders. The auxin polar transport gene AUX1 was significantly downregulated, reducing the auxin transport capacity and triggering compensatory local auxin biosynthesis. GA metabolism and signaling were globally repressed, and their responsiveness to ABA was much weaker than in the wild type. The ethylene signaling pathway represented by the ERF gene was constitutively activated and insensitive to ABA-mediated inhibition. Meanwhile, the basal expression levels of the JA biosynthesis and response genes were reduced, and these pathways failed to be properly activated under ABA treatment. The loss of *ZmABA8ox1b* function leads to the hyperactivation of the BR signaling response under ABA. Collectively, these results demonstrate that the loss of *ZmABA8ox1b* disrupts the synergistic and antagonistic interactions among hormones, leading to severe disorder in the entire regulatory network. *ZmABA8ox1b* therefore functions not only as an ABA-catabolic enzyme but also as a key node in the hormonal crosstalk that maintains ABA homeostasis and coordinates the sequential activation in multiple hormonal pathways to ensure germination.

**Figure 9:**
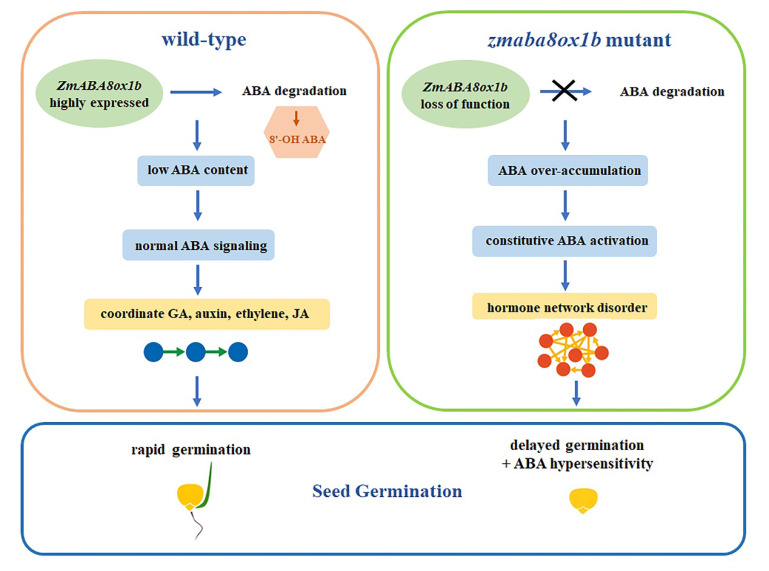
Proposed model for the regulation on seed germination by *ZmABA8ox1b.*

Based on all the results, we propose an integrated regulatory model whereby *ZmABA8ox1b* is highly expressed during early germination to accelerate ABA degradation, reduce embryonic ABA levels, release ABA-mediated inhibition, properly activate ABA signaling, and coordinate the GA, auxin, ethylene, JA, and BR pathways, ultimately promoting radicle protrusion and rapid, uniform germination. In the *ZmABA8ox1b* mutant, blocked ABA catabolism causes excessive ABA accumulation, constitutive ABA signaling activation, and a hormonal network imbalance, resulting in delayed germination and ABA hypersensitivity (Figure 9).

## 4. Materials and Methods

### 4.1. Plant Materials and Seed Germination

The CRISPR-Cas9 mutants of *ZmABA8ox1b* were generated from our previous study with B73 background [11]. For the germination phenotyping of inbred line seeds, 100 maize core inbred lines, which were collected in advance by our laboratory, were used as experimental materials (Supplementary Table S1). Uniformly mature, plump, and size-consistent maize seeds were selected. For each genotype, 50 seeds per replicate were used, with 3 biological replicates performed. Seeds were placed in 9 cm glass Petri dishes lined with 2–3 layers of filter paper with the embryo facing downward, and then incubated in the dark at 28 °C in a constant-temperature incubator, with filter paper kept moist throughout the experiment. After 20 h of seed imbibition, the number of germinated seeds was recorded every 4 h. Seed germination was defined as visible radicle protrusion (rupture of the seed coat). Cumulative germination rate was calculated as the number of germinated seeds at each time point divided by the total number of tested seeds. Seed germination rate was estimated using the time required for 50% of seeds to germinate (T_50_) [20], calculated as follows: T_50_ = ti + (tj − ti) × (N/2 − ni)/(nj − ni). This index uses the weighted mean between hours ti and tj and the cumulative seed counts (ni and nj) adjacent to half of the total sum of germinated seeds (N/2) so that ni < N/2 < nj. For ABA treatment, 50 dry maize seeds were used per biological replicate and soaked in 200 μM ABA (Sigma-Aldrich, St. Louis, MO, USA) solution, with distilled water treatment as the control. Germination was recorded every 4 h after 20 h of imbibition as described above. All experiments were performed with three biological replicates.

### 4.2. Quantification of Endogenous ABA

At 16 h after seed imbibition, embryos were isolated from seeds, immediately ground into fine powder in liquid nitrogen, and used for ABA quantification. All measurements were performed with three biological replicates. Then, 100 mg of powdered sample was extracted with 800 μL pre-chilled methanol, vortexed for 30 s, and incubated overnight at 4 °C. After centrifugation at 9000 *g* for 20 min at 4 °C, 700 μL of the supernatant was collected and vacuum-dried. Prior to mass spectrometry analysis, the dried residue was re-dissolved in 200 μL of 75% acetonitrile aqueous solution, followed by centrifugation at 14,000 *g* for 15 min at 4 °C. The resulting supernatant was subjected to instrumental analysis. For ABA quantification, standard stock solution was prepared by dissolving authentic ABA standard in methanol, and a series of gradient working solutions were further prepared to establish the standard calibration curve. The endogenous ABA content was calculated using the standard curve method with Skyline software. No isotopically labeled internal standard was used in this detection. Chromatographic separation was performed using a mobile phase consisting of solvent A (5% acetonitrile in water containing 20 mM ammonium acetate, pH 9.45) and solvent B (100% acetonitrile). Samples were maintained at 4 °C in the autosampler. The flow rate was set at 400 µL/min with an injection volume of 5 µL. The elution gradient was programmed as follows: 0–0.1 min, 90% B; 1–5 min, linear decrease from 90% to 55% B; 5–6 min, linear decrease from 55% to 40% B; 6–7.7 min, 40% B; 7.7–7.8 min, linear increase from 40% to 90% B; and 7.8–10 min, 95% B. Mass spectrometric analysis was carried out on a 5500 QTRAP system (AB SCIEX) operated in negative ion mode. The APCI source parameters were set as follows: Source Temperature, 550 °C; Ion Source Gas 1 (GAS1), 40; Ion Source Gas 2 (GAS2), 50; Curtain Gas (CUR), 25; and Nebulizer Current (NC), 3.0.

### 4.3. RNA Extraction and Quantitative Reverse-Transcription PCR

Total RNA was extracted using a Plant Total RNA Purification Kit (polysaccharide- and polyphenol-rich samples) (Tiangen Biotech, Beijing, China) according to the manufacturer’s instructions. Quantitative real-time PCR (qRT-PCR) was performed as previously described [21]. In brief, approximately 2 μg of total RNA from each sample was reverse-transcribed into first-strand cDNA using a reverse-transcription kit (TaKaRa, Kusatsu, Japan) following the manufacturer’s protocol. The qRT-PCR amplification was conducted on a CFX96 Real-Time PCR Detection System (Bio-Rad Laboratories, Inc., Hercules, CA, USA) with SYBR Green qPCR Master Mix (TaKaRa, Kusatsu, Japan). *ZmActin1* (*GRMZM2G126010*) was used as the internal reference gene. Gene-specific primer sequences used for transcript quantification are listed in Supplementary Table S6.

### 4.4. Transcriptome Sequencing and Expression Profiling

Three independent biological replicates were performed for each treatment group in RNA-seq analysis. Total RNA was extracted using the TRIzol reagent kit (Invitrogen, Carlsbad, CA, USA) according to the manufacturer’s protocol. RNA quality was assessed using an Agilent 2100 Bioanalyzer (Agilent Technologies, Palo Alto, CA, USA) and verified by RNase-free agarose gel electrophoresis. The mRNA was fragmented into short segments using fragmentation buffer and reverse-transcribed into cDNA with random primers. Second-strand cDNA was synthesized using DNA polymerase I, RNase H, dNTPs, and reaction buffer. The cDNA fragments were purified with the QiaQuick PCR extraction kit (Qiagen, Venlo, The Netherlands), end-repaired, A-tailed, and ligated to Illumina sequencing adapters. The ligation products were size-selected by agarose gel electrophoresis, amplified by PCR, and sequenced on an Illumina Novaseq 6000 platform by Gene Denovo Biotechnology Co. (Guangzhou, China).

Raw reads obtained from the sequencing platform were filtered to generate high-quality clean reads using fastp (version 0.18.0) [22] with strict filtering criteria, including removal of reads containing adapters, reads with more than 10% unknown nucleotides (N), and low-quality reads in which more than 50% of bases had a Q-value ≤ 20. The short clean reads were aligned to the ribosomal RNA (rRNA) database using Bowtie2 (version 2.2.8) [23], and reads mapped to rRNA were discarded to obtain final clean reads for subsequent assembly and quantification. Paired-end clean reads were mapped to the reference genome using HISAT2 version 2.2.4 [24]. Mapped reads for each sample were assembled using StringTie v1.3.1 [25,26] with a reference-based approach, and gene expression abundance was quantified as FPKM (Fragments Per Kilobase of transcript per Million mapped reads) using RSEM software [27] to eliminate the interference of gene length and sequencing data volume. DEGs were analyzed using two robust statistical methods for enhanced reliability. DESeq2 was applied for multi-group comparisons and overall transcriptome variation analysis, while edgeR was used for pairwise comparisons between two independent samples [28]. Genes or transcripts with a false discovery rate (FDR) below 0.05 and an absolute fold change ≥ 2 were defined as DEGs [29]. Gene Ontology (GO) enrichment analysis was performed to annotate the functional categories of DEGs, where all DEGs were mapped to GO terms in the Gene Ontology database, and significantly enriched GO terms were screened using the hypergeometric test with FDR ≤ 0.05 as the threshold, covering molecular function, cellular component, and biological process ontologies [30]. KEGG pathway enrichment analysis was further conducted to identify significantly enriched metabolic pathways and signal transduction pathways in DEGs against the whole genome background, using the same statistical method as GO analysis and a threshold of FDR ≤ 0.05 [31].

### 4.5. Statistical Analysis

All experiments were performed with at least three independent biological replicates. Statistical analysis was conducted using SPSS 26.0 software (IBM Corporation, Armonk, NY, USA) and Microsoft Excel. Data are presented as the mean ± standard error. Significant differences were determined using Student’s *t*-test (*p* < 0.05). For multiple comparisons, one-way analysis of variance (ANOVA) followed by Duncan’s multiple range test (*p* < 0.05) was performed. Pearson correlation analysis was used to evaluate the correlation.

## 5. Conclusions

This study clarified the physiological and molecular mechanisms of *ZmABA8ox1b* regulating maize seed germination. This gene facilitates rapid and uniform seed germination by promoting ABA degradation, maintaining hormone homeostasis, and coordinating multiple-phytohormone-signal crosstalk, while its functional deficiency leads to ABA accumulation, hormonal network disorder, and suppressed germination. These findings enrich the hormonal regulation theory of crop seed germination and provide promising gene resources and molecular markers for maize breeding for high seed vigor and stress resistance. The further optimization of this gene will help cultivate elite maize varieties to support stable and high-yield production.

## Figures and Tables

**Table 1:** Functional annotation of hormone-related genes from transcriptomic profiling.

Plant Hormone	Gene ID	Functional Annotation
ABA	*Zm00001d033222*	viviparous14
	*Zm00001d042460*	ABI3-VP1-transcription factor 31
	*Zm00001d003659*	SnRK2 serine threonine protein kinase7
	*Zm00001d011195*	Probable protein phosphatase 2C 74
	*Zm00001d052018*	DBP-transcription factor 1
	*Zm00001d027740*	rab30-responsive to abscisic acid30
Auxin	*Zm00001d025005*	Yucca2
	*Zm00001d030310*	auxin import carrier1
	*Zm00001d018024*	PIN-formed protein2
	*Zm00001d032094*	SAUR33-auxin-responsive
	*Zm00001d008957*	Indole-3-acetic acid-amido synthetase GH3.6
GA	*Zm00001d041660*	phytase1
	*Zm00001d041670*	phytase2
Ethylene	*Zm00001d050861*	EIL-transcription factor 5
	*Zm00001d039575*	1-aminocyclopropane-1-carboxylate oxidase
	*Zm00001d051340*	Ethylene-responsive transcription factor ERF105
	*Zm00001d021687*	Protein EIN4
JA	*Zm00001d041663*	Jasmonate-induced protein
	*Zm00001d026062*	GDSL-like Lipase/Acylhydrolase
BR	*Zm00001d022227*	MYB-transcription factor 70 (fused leaves1)
	*Zm00001d039065*	G-box binding factor

## Data Availability

The original contributions presented in this study are included in the article and Supplementary Materials. The RNA-seq raw data reported in this study are publicly available in the National Center for Biotechnology Information Sequence Read Archive (NCBI SRA, https://www.ncbi.nlm.nih.gov/sra (accessed on 20 May 2026)) under the accession number PRJNA1468289.

## Supplementary Materials

The following supporting information can be downloaded at: https://www.mdpi.com/article/10.3390/plants15111685/s1, Table S1: Seed germination T_50_ among different maize inbred lines; Table S2: List of DEGs in the WT_H_2_O vs *zmaba8ox1b*_H_2_O comparison; Table S3: List of DEGs in the WT_ABA vs *zmaba8ox1b*_ABA comparison; Table S4: List of DEGs in the WT_H_2_O vs WT_ABA comparison; Table S5: List of DEGs in the *zmaba8ox1b*_H_2_O vs *zmaba8ox1b*_ABA comparison; Table S6: Gene-specific primer pairs used in this study.
