# Supplementary material for: Functional Analysis of ZmABA8ox1b in Regulating Maize Seed Germination via ABA Catabolism and Multi-Hormone Signaling Crosstalk
Source: Plants (Basel). 2026 May 29;15(11):1685. doi: 10.3390/plants15111685 (PMC13258992; doi:10.3390/plants15111685)
Supplement: Supplementary file 1 [file plants-15-01685-s001.zip › Table S1 and Table S6.pdf]

Table S1. Seed germination T_{50} among different maize inbred lines

Number	Inbred line	T_{50}	Number	Inbred line	T_{50}
1	Ningchen07	33.49±0.48	51	7922	22.84±0.10
2	PH2VK	26.99±0.41	52	CN9127 ♀	34.74±0.23
3	PH5AD	32.38±0.38	53	CN9127 ♂	25.58±0.56
4	PH6WC	32.72±0.96	54	Xin5008	26.83±0.39
5	PH6JM	28.09±0.69	55	PH6WC	24.00±0.51
6	PH4CV	26.89±1.97	56	Zhong106	45.06±2.55
7	8085	31.85±1.00	57	Liao6049	33.60±0.35
8	Ji63	32.41±0.28	58	Zheng58	39.98±1.68
9	Dan9046	32.03±1.05	59	Chang7-2	23.60±0.43
10	Shen139	28.52±0.79	60	Liao7980	41.61±1.23
11	Shen135	26.74±0.13	61	Liao2386	26.49±0.26
12	Qi319	35.60±1.94	62	S121	37.23±0.61
13	P138	37.94±0.41	63	C8605-2	51.77±0.23
14	Ye478	36.99±0.81	64	Liao511	23.29±0.33
15	Gaishen151	25.84±0.60	65	Liao68	29.38±0.55
16	H540	30.28±0.63	66	K12	35.69±0.35
17	BM	34.61±2.03	67	Liao2345	29.81±1.11
18	C531	31.23±1.58	68	Huangzao4	37.63±0.12
19	Wu9086	25.13±0.07	69	Liao1412	33.36±1.68
20	M731	31.56±0.80	70	Liao6027	36.53±0.74
21	SA-4	31.92±1.85	71	Liao6082	33.79±1.22
22	X201	33.98±0.76	72	Liao2309	34.51±0.41
23	Da9195	27.09±0.14	73	Liao88	32.62±0.33
24	Shenyu 21 and 22 ♀	33.44±0.89	74	Shen137	39.14±0.06
25	Zi21	39.66±0.00	75	PH4CV	26.55±0.47
26	D10 ♀	28.38±0.65	76	R1	30.28±0.28
27	Liangyu 2 ♂	36.64±0.42	77	R2	26.03±0.85
28	Liangyu 2 ♀	33.47±1.06	78	R3	30.76±0.65
29	Liangyu 88 ♀	30.92±0.58	79	R5	26.66±0.23
30	C1028	37.86±0.59	80	R6	28.52±0.86
31	C279	30.42±0.59	81	R7	25.18±0.60
32	C7	25.93±0.11	82	R8	24.15±0.42
33	CG311	22.59±0.10	83	R9	27.93±0.23
34	Dan1324-1	32.28±0.17	84	A1	28.02±0.64
35	WTC	27.02±0.97	85	A2	30.14±0.27
36	Weike 606 ♂	29.78±1.77	86	A3	29.96±0.52
37	ZYH-10	24.46±0.39	87	A4	26.90±0.39
38	ZYH-9	39.56±0.19	88	A5	39.79±2.74
39	F7584	28.39±0.06	89	A7	30.87±0.44
40	ZYH-7	30.37±0.23	90	A8	24.65±0.16
41	Dika 518	28.40±0.88	91	A10	35.89±1.20
42	Qinlong 18 ♀	30.37±1.18	92	A11	23.77±0.65

43	Zhongzhong 8 ♂	28.57±1.71	93	A12	23.02±0.16
44	Weike 606 ♀	23.27±0.56	94	A13	31.51±0.18
45	ZYH-5	23.04±0.11	95	W1	24.41±0.63
46	Dan599	24.53±0.56	96	W3	23.27±0.25
47	Jing24	24.25±0.27	97	W4	27.87±1.09
48	9010	37.88±0.08	98	W5	34.94±0.96
49	H21	30.81±0.82	99	W6	28.41±0.53
50	Dan598	28.16±0.65			

Table S6. Gene-specific primer pairs used in this study.

Name	Sequence
ZmABA8ox1b-F	5′-ATGCTCGTGCTCTTCCACCACCT-3′
ZmABA8ox1b-R	5′-GGAAGCGGTTTTTCGCGTTCCTGG-3′
ZmVP1-F	5′-AGAAGGTGCTGAAGCAGAGC-3′
ZmVP1-R	5′-CTGTACCGCATGTTCCACAC-3′
ZmActin1-F	5′-CGATTGAGCATGGCATTGTCA-3′
ZmActin1-R	5′-CCCACTAGCGTACAACGAA-3′
